# Diagnostic Value of Ocular Hemodynamics and Choroidal Thickness in Unilateral Sudden Sensorineural Hearing Loss: Non-Invasive Biomarkers of Systemic Microvascular Disease

**DOI:** 10.3390/diagnostics16121903

**Published:** 2026-06-19

**Authors:** Hüseyin Findik, Muhammet Kaim, Feyzahan Uzun, Murat Okutucu, Metin Çeliker, Fatma Beyazal Çeliker, Merve Solak

**Affiliations:** 1 Department of Ophthalmology, School of Medicine, Recep Tayyip Erdogan University, Rize 53100, Turkey; muhammet.kaim@erdogan.edu.tr (M.K.); feyzahan@gmail.com (F.U.); murat.okutucu@erdogan.edu.tr (M.O.); 2 Department of Otorhinolaryngology, School of Medicine, Recep Tayyip Erdogan University, Rize 53100, Turkey; metin.celiker@erdogan.edu.tr; 3 Department of Radiology, School of Medicine, Recep Tayyip Erdogan University, Rize 53100, Turkey; fabece@yahoo.com (F.B.Ç.); merve_solak23@erdogan.edu.tr (M.S.)

**Keywords:** sudden sensorineural hearing loss, choroidal thickness, ocular hemodynamics, color Doppler ultrasonography, optical coherence tomography, systemic vasculopathy, biomarker

## Abstract

**Background/Objectives:** Although vascular mechanisms are increasingly implicated in the etiology of sudden sensorineural hearing loss (SSNHL), the inability to directly visualize the labyrinthine artery remains a diagnostic obstacle. Sharing embryological and physiological parallels with the inner ear, the eye represents an accessible surrogate organ capable of reflecting systemic microvascular status. This study aimed to evaluate the diagnostic value of ocular hemodynamic and structural parameters in patients with acute unilateral idiopathic SSNHL. **Methods:** This prospective, comparative, cross-sectional study enrolled 30 patients with acute unilateral idiopathic SSNHL and 25 age and sex matched healthy controls. Three groups were defined: the affected eye, the contralateral eye, and the control eye. Retrobulbar hemodynamics (PSV, EDV, RI, PI) were assessed by color Doppler imaging; peripapillary choroidal thickness, RNFL, GCC+, and macular thickness by swept-source OCT; and macular microvascular perfusion by OCT angiography. **Results:** End diastolic velocity in the posterior ciliary arteries was significantly reduced in both patient eye groups relative to controls (*p* < 0.001), while RI and PI were significantly elevated (*p* = 0.001 and *p* = 0.004, respectively). Comparable hemodynamic impairment was observed in the ophthalmic artery. Peripapillary choroidal thickness was bilaterally reduced in the inferior and temporal quadrants in both patient groups (*p* = 0.003 and *p* = 0.010). No significant difference was detected between affected and contralateral eyes in any parameter. RNFL, GC+, and macular thickness remained comparable across all groups. **Conclusions:** The bilateral symmetry of hemodynamic impairment and choroidal thinning suggests that SSNHL arises against a background of systemic microvascular disease. The combined use of OCT and color Doppler ultrasonography holds clinical potential as a non-invasive biomarker panel for defining the vascular phenotype of the condition.

## 1. Introduction

Sudden sensorineural hearing loss (SSNHL) is when 30 dB or more of sensorineural hearing across at least three consecutive audiometric frequencies is lost within 72 h. It is treated as an otological emergency [1,2,3,4,5]. SSNHL has an annual incidence of 5 to 27 cases per 100,000 people, making it a constant problem for doctors and patients [6,7]. In about 90% of cases, a clear cause is still unknown. Some possible causes are viral infection, autoimmune dysregulation, rupture of the intracochlear membrane, and neoplastic processes [8,9,10]. Among these, vascular pathology has attracted particular attention as the mechanism most consistent with the abruptness of onset and the severity of functional loss [8,11].

The cochlea’s vulnerability to ischemia reflects its exceptionally high metabolic demand. Any disruption in cochlear perfusion can rapidly render functional deficits irreversible [12,13,14]. This vulnerability is compounded by the anatomy of the labyrinthine artery, which functions as a terminal branch with virtually no collateral supply. Occlusion, vasospasm, or a drop in perfusion pressure therefore carries an immediate risk of cochlear ischemia [15]. The frequent co-occurrence of SSNHL with systemic microvascular conditions such as hypertension and endothelial dysfunction lends further support to a vascular etiology [8,16,17]. However, the small caliber of the labyrinthine artery and its deep course within the petrous temporal bone render direct, non-invasive flow measurement in vivo technically impossible. This anatomical constraint has directed attention toward reliable surrogate markers capable of reflecting the systemic microvascular state.

The human eye shares embryological, anatomical, and physiological parallels with inner ear circulation and offers a uniquely accessible, non-invasive window into the microvasculature [18,19]. The aim of this study was to systematically compare ocular vascular and structural parameters between patients with acute unilateral idiopathic SSNHL and age- and sex-matched healthy controls. To this end, retrobulbar hemodynamics (peak systolic velocity, end-diastolic velocity, resistive index, pulsatility index) were assessed by color Doppler imaging [20]; chorioretinal structural parameters (macular thickness, ganglion cell complex thickness, retinal nerve fiber layer thickness, peripapillary choroidal thickness) were assessed by optical coherence tomography [21,22]; and macular microvascular perfusion was assessed by OCT angiography. Furthermore, by examining whether observed changes were confined to the ipsilateral eye or present bilaterally, we investigated whether the underlying pathology was localized or systemic in nature.

## 2. Materials and Methods

This was a prospective, comparative, cross-sectional, observational study. The protocol was approved by the Non Interventional Clinical Research Ethics Committee of Recep Tayyip Erdoğan University and conducted in accordance with the principles of the Declaration of Helsinki. The study was supported by the university’s scientific research unit under grant number 2047. Written informed consent was obtained from all participants prior to enrollment.

The study population was divided into three groups: Group 1 comprised the affected eyes of patients with acute unilateral idiopathic SSNHL; Group 2 comprised the contralateral eyes of the same patients; and Group 3 comprised a randomly selected eye from each age- and sex-matched healthy control subject. This three-group design was deliberately chosen to examine whether ocular findings in SSNHL are lateralized or reflect a systemic process.

### 2.1. Participants

The SSNHL group included patients presenting within 72 h of symptom onset who met the criterion of ≥30 dB hearing loss across at least three consecutive frequencies on pure-tone audiometry (Interacoustics, Middelfart, Denmark). A diagnosis of idiopathic SSNHL was established after thorough otological evaluation and appropriate investigations excluded identifiable causes. Controls were recruited from healthy volunteers with no history of otological, neurological, or clinically significant ophthalmological disease.

The exclusion criteria applied to all participants were history of retinal pathology (diabetic retinopathy, age-related macular degeneration), optic neuropathy (including glaucoma), media opacity sufficient to degrade image quality (cataract grade >1), high refractive error (spherical equivalent >±3.0 D or astigmatism >3.0 D), prior ocular trauma or surgery, systemic inflammatory or autoimmune disease, hypertension, and other systemic vascular conditions including carotid artery stenosis and stroke.

All participants underwent a standardized ophthalmological assessment comprising best corrected visual acuity, biomicroscopic examination, intraocular pressure measurement by Goldmann applanation tonometry, and dilated fundus examination.

### 2.2. Optical Coherence Tomography

All structural and microvascular measurements were obtained using a swept-source OCT device (Topcon DRI Triton, Topcon Corporation, Tokyo, Japan) by a single experienced technician in a darkened room, without pharmacological pupil dilation unless clinically necessary. To eliminate the effect of diurnal variation on choroidal thickness, all SS-OCT scans and color Doppler ultrasonography examinations were consistently performed between 08:30 and 10:30 AM. Only scans with a quality score >5/10 were included in the analysis, and no participants or eyes were excluded from the final dataset due to this criterion. The imaging protocol comprised a 12 × 9 mm three-dimensional macular scan for automated measurement of macular thickness, ganglion cell complex (GCC+) thickness, and retinal nerve fiber layer (RNFL) thickness across the central 1, 3, and 6 mm ETDRS subfields; a peripapillary circular scan for mean and sector-specific (superior, inferior, nasal, temporal) RNFL and choroidal thickness values; and fovea-centered OCT angiography scans for superficial and deep capillary plexus vessel densities and foveal avascular zone area.

### 2.3. Color Doppler Ultrasonography

Retrobulbar hemodynamic assessments were performed by a single experienced radiologist using a Samsung V8 ultrasound device equipped with a high-frequency linear transducer (7.5–12 MHz; Samsung Medison Co., Seoul, Republic of Korea). The radiologist was completely blinded to the affected side and the clinical audiometric data of the patients to prevent observer bias. Patients were examined in the supine position with eyes gently closed; the probe was applied to the temporal aspect of the upper eyelid using sterile coupling gel. The ophthalmic artery (OA), central retinal artery (CRA), and posterior ciliary arteries (PCAs) were identified by their anatomical location and characteristic spectral waveforms. During all measurements, the Doppler angle of incidence was kept below 60 degrees, and the angle correction cursor was manually aligned parallel to the presumed axis of the vessel to secure accurate absolute velocity readings. Peak systolic velocity, end diastolic velocity, resistive index, and pulsatility index were recorded for each vessel.

### 2.4. Statistical Analysis

All analyses were performed using SPSS version 29.0 (IBM Corp., Armonk, NY, USA). The normality of continuous variables was assessed with the Shapiro–Wilk test; normally distributed data are presented as mean ± standard deviation. Generalized Linear Mixed Models analysis was performed to compare the three groups. Generalized Linear Mixed Models analysis was applied to account for the non-independence of two eyes from the same patient (intra-individual correlation). This approach provided more reliable results by statistically adjusting for the correlation between the right and left eyes of the same patient. Categorical variables were compared using the chi-square test. Pearson correlation analysis was used to examine relationships between variables reaching statistical significance. Sample size was determined by an a priori power analysis targeting a medium-to-large effect size (Cohen’s d = 0.7) with 80% power (β = 0.20) at a significance level of α = 0.05, yielding a minimum required sample of 52 participants. A *p*-value below 0.05 was considered statistically significant.

## 3. Results

A total of 85 eyes from 55 individuals were included in the final analysis: 30 affected and 30 contralateral eyes from 30 patients with acute unilateral idiopathic SSNHL and 25 eyes from 25 healthy controls. The groups were well matched for age and sex (both *p* > 0.05). Furthermore, baseline intraocular pressure (IOP) and systemic blood pressure parameters (mean arterial pressure) recorded at the time of examination showed no statistically significant differences between the patient and control groups (all *p* > 0.05), establishing a uniform baseline for ocular perfusion pressure. Additionally, baseline body mass index (BMI) and smoking status were evaluated, showing no statistically significant differences between the patient and control groups (all *p* > 0.05) (Table 1).

Structural Neural Parameters: No statistically significant differences were found among the three groups in retinal neural layer measurements. Mean RNFL thickness was 106.90 ± 9.81 µm in affected eyes, 107.43 ± 10.29 µm in contralateral eyes, and 107.24 ± 8.88 µm in control eyes (*p* = 0.977). Macular thickness and GCC+ thickness across all ETDRS subfields were likewise comparable between groups (all *p* > 0.05) (Table 2, Table 3 and Table 4).

Peripapillary Choroidal Thickness: The analysis of peripapillary choroidal thickness (PCT) revealed significant structural differences among the groups, specifically in the inferior and temporal quadrants. The inferior choroid was significantly thinner in both the affected eyes (123.37 ± 52.13 µm; *p* = 0.002) and the contralateral eyes (138.57 ± 66.98 µm; *p* = 0.034) of SSNHL patients compared to the healthy control group (182.12 ± 66.85 µm). Similarly, the temporal choroid exhibited a significant reduction in thickness in both the affected (134.10 ± 48.55 µm; *p* = 0.016) and contralateral eyes (137.57 ± 43.60 µm; *p* = 0.033) relative to controls (172.64 ± 57.68 µm). Notably, there were no statistically significant differences between the affected and contralateral eyes of the patients in any of the four quadrants (all *p* = 1.000), indicating a bilateral and symmetrical thinning, while the superior (*p* = 0.942) and nasal (*p* = 0.842) quadrants remained unaffected and stable across all three groups (Table 5).

OCT Angiography: There were no significant differences in vessel densities in the superficial and deep capillary plexuses and the foveal avascular zone area among the three groups (all *p* > 0.05) (Table 6).

### Color Doppler Imaging

Color Doppler imaging showed that the most significant hemodynamic changes were in the posterior ciliary arteries (PCAs). End-diastolic velocity (EDV) in the posterior ciliary arteries (PCAs) was markedly diminished in both affected (8.82 ± 5.05 cm/s) and contralateral (7.79 ± 4.53 cm/s) eyes when compared to controls (14.63 ± 6.39 cm/s; *p* < 0.001). Resistive index (RI) and pulsatility index (PI) were correspondingly elevated in both patient eye groups relative to controls (*p* = 0.001 and *p* = 0.004, respectively). A pattern of increased vascular resistance was similarly evident in the ophthalmic artery (OA), with significant group differences in both RI (*p* = 0.004) and PI (*p* = 0.008). In the central retinal artery (CRA), a significant difference was detected for PI alone (*p* = 0.028) (Table 7). Post hoc pairwise comparisons reinforced the bilateral nature of hemodynamic impairment. For the PCAs, both affected (RI: 0.72 ± 0.10; *p* = 0.005) and contralateral (RI: 0.72 ± 0.10; *p* = 0.003) eyes exhibited significantly higher resistance than controls (RI: 0.63 ± 0.11). This finding was reproduced in the OA, where RI was significantly elevated in both patient eye groups compared with controls (*p* = 0.015 and *p* = 0.007, respectively).

**Figure 1:**
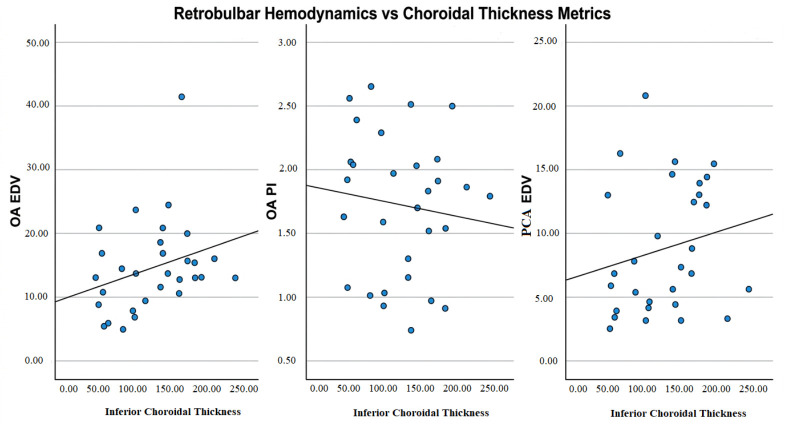
Scatter plots demonstrating the relationship between the inferior choroidal stromal index and retrobulbar hemodynamic parameters OA EDV: Ophthalmic Artery End Diastolic Velocity; OA PI: Ophthalmic Artery Pulsatility Index; PCA EDV: Ciliary Artery End Diastolic Velocity.

Correlation Analysis: Pearson correlation analysis examining the relationship between peripapillary choroidal thickness and retrobulbar hemodynamic parameters revealed significant associations specific to the inferior quadrant. Inferior choroidal thickness correlated positively with OA EDV (r = 0.300; *p* = 0.026) and PCA EDV (r = 0.284; *p* = 0.036), and negatively with OA PI (r = −0.271; *p* = 0.045). No significant correlations were identified between temporal choroidal thickness and the hemodynamic parameters examined (Table 8; Figure 1).

## 4. Discussion

The findings of this study provide multimodal evidence supporting the hypothesis that idiopathic SSNHL, at least in a meaningful subset of patients, represents a manifestation of underlying systemic vasculopathy rather than a purely localized otological event. The cornerstone of this interpretation is the striking bilateral symmetry of the observed ocular changes. We identified significantly elevated vascular resistance and reduced diastolic perfusion in the retrobulbar circulation, most prominently in the ciliary and ophthalmic arteries, as evidenced by increased RI and PI values. Critically, these hemodynamic abnormalities were not confined to the ipsilateral eye but were replicated, to a statistically equivalent degree, in the clinically asymptomatic contralateral eye. This bilateral pattern effectively argues against an isolated thromboembolic event in the labyrinthine artery as the sole causative mechanism, and instead points toward a systemic process, such as generalized endothelial dysfunction or autonomic dysregulation, simultaneously affecting sensitive microvascular beds throughout the body [23,24,25]. Both the inner ear and the choroid represent vulnerable end-organ territories supplied by terminal arterial circulation with high metabolic demand and limited collateral reserve [26,27]. Furthermore, the eye is supplied by the ophthalmic artery via the internal carotid artery (anterior circulation), whereas the inner ear is perfused by the labyrinthine artery originating from the posterior circulation. The simultaneous presence of microvascular alterations in two distinct major circulatory systems further supports generalized systemic microvasculopathy rather than a localized thromboembolic event.

The structural findings align with the hemodynamic observations in a physiologically consistent manner. A significant and bilateral thinning of the peripapillary choroid, primarily concentrated in the inferior and temporal watershed zones, can be interpreted as the anatomical correlate of the perfusion insufficiency assessed by Doppler imaging [28]. These areas are acknowledged as the most vulnerable to decreases in perfusion pressure from the posterior ciliary arteries, and atrophy in these regions is a well-established indicator of chronic or acute-on-chronic ischemia [29,30]. The structural integrity of the retinal neural layers, such as macular thickness, GCC+, and RNFL, was also significant, as it remained similar to that of healthy controls across all assessed subfields. This phenomenon, which we term functional–structural dissociation, suggests that the acute vascular insult was sufficient to impair cochlear function in a metabolically demanding organ, yet had not reached the threshold required to trigger retinal apoptosis and measurable neuronal atrophy [31,32]. This dissociation underscores the existence of a critical therapeutic window during which interventions aimed at restoring perfusion may prevent irreversible end-organ damage, both in the cochlea and potentially in other vulnerable vascular territories [23,33].

The physiological explanation for unilateral labyrinthine infarction occurring against a background of bilateral, asymptomatic ocular changes stems from the distinct vascular anatomy and metabolic demands of the two systems. The inner ear is a terminal vascular bed; the labyrinthine artery has no collateral anastomosis, rendering the metabolically demanding cochlea highly susceptible to irreversible damage from a single localized vasospastic or microthrombotic event. Conversely, the choroid is supplied by an extensive network of multiple posterior ciliary arteries with significant collateral redundancy and advanced local autoregulation. This anatomical protection buffers the eye against sudden functional failure, allowing it to absorb systemic microvascular changes as chronic, bilateral, and asymptomatic structural thinning rather than acute infarction.

Our findings are consistent with, and extend, prior work examining the relationship between ocular circulation and SSNHL. Several studies employing color Doppler imaging have similarly reported elevated RI and PI values in the ophthalmic and posterior ciliary arteries of SSNHL patients, concluding that impaired ocular hemodynamics are associated with this condition [34,35]. The present study advances this understanding by systematically demonstrating the bilateral nature of these changes through a three-group comparative design a methodological feature not consistently employed in the existing literature. By showing that the contralateral eye is affected to a degree statistically indistinguishable from the ipsilateral eye, we provide stronger evidence for a systemic etiology than studies examining only the affected side. The choroidal thinning findings warrant particular attention as a novel contribution. Choroidal thinning is increasingly recognized as an early structural marker of systemic microvascular disease, frequently preceding clinically manifest retinopathy in conditions such as diabetes, hypertension, and chronic kidney disease [31,36,37,38]. Our quadrant specific analysis refines this observation by demonstrating that thinning is most pronounced in regions physiologically consistent with a perfusion deficit arising from the posterior ciliary arteries [28]. The utilization of swept-source OCT in this study provided a distinct practical advantage over conventional spectral-domain OCT. Its longer wavelength (1050 nm) enables deeper tissue penetration and superior visualization of the chorioscleral interface, securing highly reproducible and accurate peripapillary choroidal thickness measurements without signal attenuation. The absence of significant changes in OCT angiography data is itself informative. This finding may reflect that the primary vascular insult occurs at the level of larger feeding arterioles, the ciliary arteries, rather than at the level of the choriocapillaris or retinal capillary plexuses. Alternatively, the duration of the insult may have been insufficient to produce detectable capillary loss with current OCT-A technology [30,32].

The clinical implications of these findings operate on several levels. By demonstrating that non-invasive ophthalmic imaging can detect objective evidence of systemic vasculopathy, this study proposes a practical means of phenotyping patients beyond the generic label of “idiopathic.” The combined use of color Doppler ultrasonography and OCT may serve as an accessible, non-invasive biomarker panel for defining a vascular phenotype of SSNHL. Patients identified by this phenotype could be prioritized for treatments targeting vascular perfusion, including systemic corticosteroids, vasodilators, rheological agents, and hyperbaric oxygen therapy [16,39]. Furthermore, a confirmed hemodynamic abnormality should prompt a more thorough cardiovascular risk evaluation, given that such patients may carry elevated risk for other ischemic events, including stroke and myocardial infarction [24,25,26].

Several limitations of this study merit acknowledgment. The cross-sectional design permits the identification of associations but does not establish causality. Whether the observed vasculopathy predisposes to hearing loss, or whether both share a common systemic substrate, can only be addressed through longitudinal studies following at-risk individuals over time. Although the sample size was adequate for testing the primary hypotheses, multi-center studies will be necessary to establish the generalizability of these findings. Both imaging modalities carry a degree of operator dependency, and interobserver variability may represent a limiting factor in broader clinical application. Additionally, the lack of exact axial length measurement via routine ocular biometry is a specific limitation regarding the interpretation of choroidal thickness. Furthermore, the strict exclusion of patients with known systemic vascular diseases, while necessary to eliminate major confounders and ensure internal validity, limits the generalizability of our findings to the broader, real-world SSNHL population where vascular comorbidities are highly prevalent. Finally, while the use of the eye as a surrogate organ is physiologically well justified and supported by our data, it remains an indirect approach to assessing labyrinthine arterial flow.

This study demonstrates that acute idiopathic SSNHL is associated with bilateral ocular hemodynamic impairment and corresponding choroidal thinning in the watershed zones, while retinal neural structures remain structurally intact. The fact that these changes were present not only in the ipsilateral eye but to an equivalent degree in the clinically asymptomatic contralateral eye suggests a systemic microvascular process simultaneously affecting both eyes and, in all likelihood, the inner ear, rather than a localized arterial event.

The combined use of OCT and color Doppler ultrasonography holds clinical promise as an accessible, non-invasive, and quantifiable biomarker panel for defining the vascular phenotype of SSNHL. This phenotyping approach may allow patients to be stratified beyond the generic label of “idiopathic,” informing the selection and timing of perfusion-targeted therapies and prompting a more thorough cardiovascular risk evaluation. However, due to the cross-sectional design of this study, direct causality between the observed ocular findings and SSNHL cannot be definitively established. Longitudinal, multi-center studies are needed to establish causality, determine the prognostic value of ocular parameters in predicting hearing recovery, and evaluate the efficacy of targeted vascular interventions in this specific patient subset.

## Figures and Tables

**Table 1:** Demographic and baseline clinical characteristics of the study groups.

Parameters	Affected Eye (G1) (*n* = 30)	Contralateral Eye (G2) (*n* = 30)	Control Group (G3) (*n* = 25)	*p*-Value
Age (Mean ± SD)	51 ± 15	51 ± 15	47 ± 9	0.355 ^a^
Gender (Male/Female, N)	18/12	18/12	13/12	0.794 ^b^
Intraocular Pressure (mmHg)	14.5 ± 2.1	14.6 ± 2.0	14.2 ± 2.3	0.512 ^a^
Mean Arterial Pressure (mmHg)	92.1 ± 7.4	92.1 ± 7.4	90.8 ± 6.9	0.485 ^a^
Body Mass Index (kg/m^2^)	24.8 ± 3.2	24.8 ± 3.2	24.1 ± 2.9	0.380 ^a^
Smoking History (n, %)	10 (33.3%)	10 (33.3%)	7 (28.0%)	0.845 ^a^

SD: Standard Deviation, *n*: Number of subjects. ^a^: *p*-value calculated using Generalized Linear Mixed Models analysis. ^b^: *p*-value calculated using Chi-square test.

**Table 2:** Intergroup comparison of macular thickness (µm) across ETDRS grid.

Macular Thickness	Affected Eye (1) (Mean ± SD)	Contralateral Eye (2) (Mean ± SD)	Control Eye (3) (Mean ± SD)	*p*-Value ^a^	*p* (1 vs. 2)	*p* (1 vs. 3)	*p* (2 vs. 3)
Central 1 mm	244.60 ± 31.38	244.07 ± 30.93	241.20 ± 18.44	0.893	1.000	1.000	1.000
3 mm Superior	303.47 ± 32.96	309.40 ± 16.62	310.44 ± 17.67	0.496	1.000	0.856	1.000
3 mm Inferior	308.13 ± 18.65	307.97 ± 15.48	301.88 ± 28.26	0.470	1.000	0.825	0.863
3 mm Nasal	307.33 ± 24.33	311.53 ± 17.12	306.20 ± 28.52	0.667	1.000	1.000	1.000
3 mm Temporal	295.57 ± 22.88	294.90 ± 16.01	297.52 ± 17.37	0.873	1.000	1.000	1.000
6 mm Superior	273.23 ± 18.86	277.57 ± 18.00	274.20 ± 16.82	0.623	1.000	1.000	1.000
6 mm Inferior	265.30 ± 15.66	265.20 ± 14.54	266.84 ± 14.06	0.903	1.000	1.000	1.000
6 mm Nasal	288.73 ± 21.92	290.77 ± 16.79	287.88 ± 13.95	0.828	1.000	1.000	1.000
6 mm Temporal	259.47 ± 22.14	259.57 ± 14.13	260.72 ± 16.16	0.961	1.000	1.000	1.000

SD: Standard Deviation. ^a^: *p*-value calculated using Generalized Linear Mixed Models analysis. Pairwise comparisons were performed using Bonferroni post hoc test.

**Table 3:** Intergroup comparison of ganglion cell complex (GCC+) thickness (µm).

GCC+ Parameter	Affected Eye (1) (Mean ± SD)	Contralateral Eye (2) (Mean ± SD)	Control Eye (3) (Mean ± SD)	*p*-Value ^a^	*p* (1 vs. 2)	*p* (1 vs. 3)	*p* (2 vs. 3)
Central 1 mm	50.97 ± 4.5	49.97 ± 4.2	49.32 ± 3.8	0.902	1.000	1.000	1.000
3 mm Superior	90.60 ± 7.2	91.00 ± 6.8	91.92 ± 6.5	0.882	1.000	1.000	1.000
3 mm Inferior	91.23 ± 7.5	91.03 ± 7.1	90.24 ± 6.9	0.919	1.000	1.000	1.000
3 mm Nasal	90.00 ± 8.1	91.47 ± 7.4	91.48 ± 7.2	0.841	1.000	1.000	1.000
3 mm Temporal	86.50 ± 6.9	87.20 ± 6.5	88.00 ± 6.1	0.832	1.000	1.000	1.000
6 mm Superior	64.93 ± 5.8	66.83 ± 5.2	65.00 ± 5.4	0.529	0.945	1.000	1.000
6 mm Inferior	62.63 ± 5.4	61.73 ± 5.1	64.20 ± 4.9	0.423	1.000	1.000	0.581
6 mm Nasal	70.20 ± 6.2	70.20 ± 5.9	70.16 ± 5.7	1.000	1.000	1.000	1.000
6 mm Temporal	69.73 ± 6.5	70.43 ± 6.0	71.16 ± 5.8	0.777	1.000	1.000	1.000

GCC+: Ganglion Cell Complex, SD: Standard Deviation. ^a^: *p*-value calculated using Generalized Linear Mixed Models analysis. Pairwise comparisons were performed using Bonferroni post hoc test.

**Table 4:** Intergroup comparison of peripapillary retinal nerve fiber layer (RNFL) thickness (µm).

RNFL Parameters	Affected Eye (1) (Mean ± SD)	Contralateral Eye (2) (Mean ± SD)	Control Eye (3) (Mean ± SD)	*p*-Value ^a^	*p* (1 vs. 2)	*p* (1 vs. 3)	*p* (2 vs. 3)
Average RNFL	106.90 ± 9.81	107.43 ± 10.29	107.24 ± 8.88	0.977	1.000	1.000	1.000
Superior RNFL	133.53 ± 15.42	133.77 ± 16.72	129.88 ± 10.97	0.564	1.000	1.000	1.000
Inferior RNFL	136.67 ± 17.60	134.93 ± 17.58	135.72 ± 13.93	0.921	1.000	1.000	1.000
Nasal RNFL	85.50 ± 15.16	87.90 ± 16.23	87.56 ± 15.15	0.814	1.000	1.000	1.000
Temporal RNFL	72.10 ± 10.99	73.23 ± 11.53	75.68 ± 12.05	0.510	1.000	0.761	1.000

RNFL: Retinal Nerve Fiber Layer, SD: Standard Deviation. ^a^: *p*-value calculated using Generalized Linear Mixed Models analysis. Pairwise comparisons were performed using Bonferroni post hoc test.

**Table 5:** Intergroup comparison of peripapillary choroidal thickness (PCT) (µm).

PCT Quadrants	Affected Eye (1) (Mean ± SD)	Contralateral Eye (2) (Mean ± SD)	Control Eye (3) (Mean ± SD)	*p*-Value ^a^	*p* (1 vs. 2)	*p* (1 vs. 3)	*p* (2 vs. 3)
Superior Choroid	168.43 ± 74.82	168.73 ± 74.58	174.52 ± 66.24	0.942	1.000	1.000	1.000
Inferior Choroid	123.37 ± 52.13	138.57 ± 66.98	182.12 ± 66.85	0.003 *	1.000	0.002 *	0.034 *
Nasal Choroid	154.13 ± 63.61	150.40 ± 62.85	160.12 ± 56.22	0.842	1.000	1.000	1.000
Temporal Choroid	134.10 ± 48.55	137.57 ± 43.60	172.64 ± 57.68	0.010 *	1.000	0.016 *	0.033 *

PCT: Peripapillary Choroidal Thickness, SD: Standard Deviation. *: Statistically significant (*p* < 0.05). ^a^: *p*-value calculated using Generalized Linear Mixed Models analysis. Pairwise comparisons were performed using Bonferroni post hoc test.

**Table 6:** Intergroup comparison of macular microvascular parameters (OCTA).

Parameter	Affected Eye (1) (Mean ± SD)	Contralateral Eye (2) (Mean ± SD)	Control Eye (3) (Mean ± SD)	*p* Value ^a^	*p* (1 vs. 2)	*p* (1 vs. 3)	*p* (2 vs. 3)
SCP Vessel Density (%)							
Central	20.81 ± 6.30	19.20 ± 4.93	18.74 ± 3.54	0.287	0.685	0.424	1.000
Superior	42.07 ± 4.99	41.54 ± 5.13	40.70 ± 3.58	0.554	1.000	0.841	1.000
Inferior	41.18 ± 5.00	40.45 ± 6.32	40.17 ± 3.27	0.747	1.000	1.000	1.000
Nasal	41.15 ± 4.90	41.13 ± 4.77	40.99 ± 3.30	0.990	1.000	1.000	1.000
Temporal	43.59 ± 4.80	43.78 ± 4.34	42.41 ± 2.66	0.425	1.000	0.874	0.668
DCP Vessel Density (%)							
Central	14.97 ± 8.68	16.75 ± 9.72	15.95 ± 3.70	0.692	1.000	1.000	1.000
Superior	41.59 ± 9.75	41.94 ± 7.76	42.87 ± 4.24	0.824	1.000	1.000	1.000
Inferior	38.83 ± 10.94	39.43 ± 9.63	41.81 ± 3.85	0.439	1.000	0.661	0.982
Nasal	42.24 ± 8.34	42.87 ± 6.28	44.19 ± 3.41	0.532	1.000	0.805	1.000
Temporal	39.86 ± 8.60	40.57 ± 6.45	43.18 ± 3.61	0.169	1.000	0.212	0.459
FAZ Area (µm^2^)							
Superficial FAZ	255.49 ± 102.68	259.13 ± 95.61	266.36 ± 82.91	0.913	1.000	1.000	1.000
Deep FAZ	251.92 ± 110.91	260.62 ± 103.62	238.35 ± 95.23	0.731	1.000	1.000	1.000

SCP: Superficial Capillary Plexus, DCP: Deep Capillary Plexus, FAZ: Foveal Avascular Zone, SD: Standard Deviation. *p* value ^a^: *p*-value calculated using Generalized Linear Mixed Models analysis. Pairwise comparisons were performed using Bonferroni post hoc test.

**Table 7:** Intergroup comparison of color Doppler imaging (CDI) parameters.

RDUS Parameters	Affected Eye (1) (Mean ± SD)	Contralateral Eye (2) (Mean ± SD)	Control Eye (3) (Mean ± SD)	*p* Value ^a^	*p* (1 vs. 2)	*p* (1 vs. 3)	*p* (2 vs. 3)
Ophthalmic Artery (OA)							
PSV (cm/s)	63.02 ± 25.56	59.00 ± 15.26	61.97 ± 19.98	0.741	1.000	1.000	1.000
EDV (cm/s)	14.63 ± 7.23	14.07 ± 7.13	19.19 ± 7.33	0.022 *	1.000	0.066	0.034 *
Resistive Index	0.76 ± 0.10	0.76 ± 0.09	0.69 ± 0.09	0.004 *	1.000	0.015 *	0.007 *
Pulsatility Index	1.72 ± 0.56	1.65 ± 0.43	1.33 ± 0.38	0.008 *	1.000	0.010 *	0.041 *
Central Retinal Artery (CRA)							
PSV (cm/s)	27.59 ± 8.87	30.39 ± 8.97	26.71 ± 7.19	0.237	0.609	1.000	0.336
EDV (cm/s)	7.07 ± 3.00	8.56 ± 4.57	8.60 ± 3.03	0.195	0.348	0.375	1.000
Resistive Index	0.73 ± 0.10	0.73 ± 0.11	0.67 ± 0.09	0.069	1.000	0.095	0.180
PI	1.59 ± 0.71	1.42 ± 0.57	1.16 ± 0.31	0.028 *	0.797	0.023 *	0.301
PSA							
PSV (cm/s)	31.34 ± 14.70	28.90 ± 13.44	39.50 ± 14.83	0.022 *	1.000	0.114	0.023 *
EDV (cm/s)	8.82 ± 5.05	7.79 ± 4.53	14.63 ± 6.39	<0.001 *	1.000	<0.001 *	<0.001 *
Resistive Index	0.72 ± 0.10	0.72 ± 0.10	0.63 ± 0.11	0.001 *	1.000	0.005 *	0.003 *
Pulsatility Index	1.50 ± 0.81	1.45 ± 0.50	1.00 ± 0.29	0.004 *	1.000	0.007 *	0.016 *

PSV: Peak Systolic Velocity, EDV: End-Diastolic Velocity, PI: Pulsatility Index, SD: Standard Deviation. * Statistically significant (*p* < 0.05). *p*-Value ^a^: *p*-value calculated using Generalized Linear Mixed Models analysis. Pairwise comparisons were performed using Bonferroni post hoc test.

**Table 8:** Pearson correlation analysis between choroidal thickness and hemodynamic parameters.

Hemodynamic Parameters	Inferior PCT r (*p*)	Temporal PCT r (*p*)
Ophthalmic Artery (OA)		
End-Diastolic Velocity (EDV)	0.300 (0.026) *	0.178 (0.192)
Resistive Index (RI)	−0.258 (0.057)	0.043 (0.756)
Pulsatility Index (PI)	−0.271 (0.045) *	0.022 (0.873)
Central Retinal Artery (CRA)		
End-Diastolic Velocity (EDV)	0.191 (0.163)	0.170 (0.214)
Resistive Index (RI)	−0.229 (0.092)	−0.073 (0.597)
Pulsatility Index (PI)	−0.184 (0.179)	−0.076 (0.581)
Posterior Ciliary Arteries (PCA)		
End-Diastolic Velocity (EDV)	0.284 (0.036) *	0.142 (0.302)
Resistive Index (RI)	−0.200 (0.144)	−0.186 (0.174)
Pulsatility Index (PI)	−0.119 (0.389)	−0.076 (0.582)

r: Pearson Correlation Coefficient, PCT: Choroidal Thickness, OA: Ophthalmic Artery, CRA: Central Retinal Artery, PCA: Posterior Ciliary Arteries. *: Correlation is significant at the 0.05 level (2-tailed).

## Data Availability

The original contributions presented in this study are included in the article; further inquiries can be directed to the corresponding author.
